# Two sides of the same coin? The association between suicide stigma and suicide normalisation

**DOI:** 10.1017/S2045796022000610

**Published:** 2022-11-04

**Authors:** N. Oexle, D. Valacchi, P. Grübel, T. Becker, N. Rüsch

**Affiliations:** Department of Psychiatry II, University of Ulm and BKH Günzburg, 89073 Ulm, Germany

**Keywords:** normalisation, prevention, society, stigma, suicide

## Abstract

**Aims:**

Evidence suggests that suicide stigma (i.e. negative attitudes towards persons affected by suicide/suicidality) and suicide normalisation (i.e. liberal attitudes towards suicide) are both associated with increased suicide risk. Despite conceptual similarities and potential interaction, suicide stigma and suicide normalisation have usually been investigated separately. We used cross-sectional data from a community sample to test the association between suicide stigma and suicide normalisation as well as to identify their respective determinants and consequences.

**Methods:**

Participants were *N* = 3.269 adults recruited from an established online-panel using quotas to reflect the composition of the German general population with regard to age, gender, education and region. We collected information about suicide stigma, suicide normalisation, intentions to seek help for suicidality, current suicidality, suicide literacy, negative mood and socio-demographic variables. We used regression modelling to determine the association between suicide stigma and suicide normalisation as well as to identify their determinants and consequences.

**Results:**

Suicide stigma and suicide normalisation were inversely associated so that higher suicide stigma scores were linked to lower suicide normalisation. More suicide stigma was associated with reduced intentions to seeking professional help, increased willingness to seek help from family and friends and lower odds to experience current suicidality, however the association between suicide stigma and intentions to seek professional help diminished after controlling for confounding variables. Increased suicide normalisation was linked to reduced intentions to seek help from professionals or family and friends, as well as higher odds to experience current suicidality, even after controlling for confounding variables.

**Conclusions:**

Our findings suggest that interventions to reduce public suicide stigma are at risk to unintentionally increase suicide normalisation, which appears to be a key barrier to seeking help for suicidality. Future research should therefore identify strategies to improve attitudes towards persons affected by suicidality that avoid normalisation, i.e. do not convey the message of suicide as an acceptable solution for difficult life situations. One strategy with great potential to safely reduce public suicide sigma could be interventions that stimulate interpersonal contact with affected persons sharing their recovery story.

## Introduction

With globally around 700 000 suicides each year, suicide prevention is an important public health issue (WHO, 2019). Despite evidence for a strong link between suicide and mental illness, suicide is a complex phenomenon with various social and individual determinants (WHO, 2014). Suicide oftentimes occurs in moments of crisis and is generally linked to the experience of conflict, disaster, violence, abuse, loss and social isolation (WHO, 2021). While national suicide rates tend to be relatively stable over time, they vary greatly across cultures. For example, in 2019 the age-standardised suicide rate for Europe was 10.5 per 100 000, compared to 11.2 per 100 000 in Africa and 6.4 per 100 000 in the Eastern Mediterranean region (WHO, 2019). Interestingly, immigrants usually maintain the national suicide rates of their country of origin when moving from one place to another (Voracek and Loibl, 2008; Spallek *et al*., 2015), suggesting that cultural values about and social attitudes towards suicide contribute to observed variations in national suicide rates (Mueller *et al*., 2021). Indeed, according to the Cultural Theory and Model of Suicide, cultural meanings associated with both stressful life events as well as suicide itself are important determinants of suicidal thoughts and behaviours (Chu *et al*., 2010). Past research exploring the role of culture and social attitudes for suicidal behaviour has generally focused on one of two related concepts, namely suicide stigma (i.e. negative attitudes towards persons affected by suicide/suicidality) and suicide normalisation (i.e. liberal/accepting attitudes towards suicide).

The term stigma was first introduced by sociologist Erving Goffman, who defined it as an attribute devaluing a person ‘from a whole and usual […] to a tainted, discounted one’ (Goffman, 1963; p. 3), with such attributes being visible (e.g. skin colour) or invisible (e.g. mental illness). Whether a certain attribute is stigmatised is not random but usually serves one of three evolutionarily beneficial purposes, namely exploitation and domination (‘keep people down’), avoidance of disease (‘keep people away’) or the enforcement of social norms (‘keep people in’) (Phelan *et al*., 2008; Rüsch, 2022). Indeed, suicide is judged negatively in many cultures, and scholars have argued that suicide stigma could be a crucial target for suicide prevention (Carpiniello and Pinna, 2017; Oexle *et al*., 2020). Due to suicide stigma, people who attempted suicide are often seen as cowards, selfish or losers (Sheehan *et al*., 2017*b*), while persons who lost a loved one to suicide are commonly perceived as guilty, broken or pitiful (Sheehan *et al*., 2018) with detrimental effects for their life opportunities and mental health. The perception of suicide stigma was associated with increased distress and suicidality among persons who survived a suicide attempt (Mayer *et al*., 2020) and those who lost a loved one to suicide (Oexle *et al*., 2020), which are both high risk groups for suicide. Furthermore, suicide stigma has been reported as a barrier for seeking help when suicidality is experienced (Calear *et al*., 2014; Han *et al*., 2018). Noteworthy, despite conceptual similarities, existing evidence suggests that suicide stigma and mental illness stigma are two distinct concepts (Rimkeviciene *et al*., 2015; Sheehan *et al*., 2017*a*, 2017*b*). For example, Sheehan *et al*. (2017*b*) found that while people who have a mental illness or experience suicidality were both judged as dangerous or incompetent, the stereotypes ‘selfish’ and ‘immoral’ only applied to people who experience suicidality.

Based on existing literature, interventions to improve public attitudes towards persons affected by suicidality/suicide can contribute to suicide prevention. However, as outlined above, one core purpose of suicide stigma may be the enforcement of social norms (i.e. ‘to keep people in’) that could serve as a barrier to suicidal behaviour. Indeed, initial evidence suggests that while negative attitudes towards persons affected by suicide/suicidality (i.e. suicide stigma) are harmful for suicide prevention, liberal/accepting attitudes towards suicide in general (i.e. suicide normalisation) might increase suicide risk. According to suicide-scripts theory (Canetto, 2021), suicidal behaviour is most likely when it is socially accepted or even expected for certain persons in certain situations, what at least partially explains elevated suicide rates in certain regions (Pepper, 2017) or among certain social groups such as the elderly (Winterrowd *et al*., 2017), youth (Kleiman, 2015) and sexual minorities (Canetto *et al*., 2020). In line with that, several studies observed increased national suicide rates in countries with more liberal attitudes towards suicide (Stack and Kposowa, 2008; Eskin *et al*., 2016). Phillips and Luth (2020) used representative longitudinal data from the US and found liberal attitudes towards suicide to predict personal suicide risk later in life. Finally, one study compared data from 12 culturally diverse countries and observed the highest suicide rates in countries with negative attitudes towards suicidal persons but liberal attitudes towards suicidal behaviour (Eskin *et al*., 2016), suggesting that both suicide stigma and suicide normalisation can be harmful for suicide prevention.

Based on these findings, we believe that the distinction between attitudes towards people (i.e. suicide stigma) *v*. attitudes towards behaviour (i.e. suicide normalisation) is crucial when aiming to understand how social attitudes about suicide impact suicide risk. However, despite great relevance for suicide prevention, the relationship between suicide stigma and suicide normalisation as well as their determinants (i.e. factors that influence their levels) and consequences (i.e. factors that are influenced by their levels) remain incompletely understood. This study aims to close this knowledge gap and investigates associations between suicide stigma, suicide normalisation, and various potential correlates relevant for suicide prevention. Based on existing literature outlined above, we expected an inverse association between suicide stigma and suicide normalisation. Additionally, we expected both suicide stigma and suicide normalisation as potentially harmful for suicide prevention, i.e. to be associated with decreased intentions to seek help and increased suicidality.

## Method

### Procedure and participants

This paper is based on baseline data derived from the RISE study (RISE: Reducing public suicide stigma), a multi-arm online randomised controlled trial testing the efficacy of contact and education-based interventions to reduce public suicide stigma. A manuscript focusing on intervention evaluation is currently being prepared (Oexle *et al*., Manuscript in preparation). Participants were recruited from a German research online panel (Respondi mingle) and all data collection (incl. randomisation & interventions) took place on the online research platform SoSci Survey (www.soscisurvey.de) between February and March 2021. Quotas were applied to ensure that the recruited sample represented the composition of the German general population with regard to age, gender, education and region. Participants had to be at least 18 years old and indicate no current suicide risk based on answering ‘no’ to the following item adapted from the PHQ-9 (Kroenke *et al*., 2001): ‘During the last three months, did you oftentimes think that you would be better off dead?’ Persons who responded with ‘yes’ were excluded and received digital information about mental health services as well as were offered to talk to a psychiatrist (NR). The study was registered online (clinicaltrials.gov; NCT04756219) and approved by the ethical review board of Ulm University (reference number: 352/20). At baseline *N* = 4.418 persons provided online informed consent and were screened for eligibility. A total of 3.897 persons met the inclusion criteria and 3.789 participants completed the baseline questionnaires.

### Questionnaires

Suicide stigma (i.e. negative attitudes towards persons who survived a suicide attempt) was measured using the German version of the 8-item stigma subscale of the Stigma of Suicide Scale – Short Form (Batterham *et al*., 2013; Ludwig *et al*., 2020). Participants rated their agreement with eight negative one-word descriptors (e.g. immoral) of persons who attempted suicide using a 5-point Likert scale (1/strongly disagree, 2/disagree, 3/neutral, 4/agree, 5/strongly agree). We calculated mean scores across all items with higher scores reflecting more suicide stigma (Cronbach's *α* = 0.88).

Suicide normalisation (i.e. liberal attitudes towards suicide) were measured by the German version of the 8-item right to commit suicide subscale of the Cognitions Concerning Suicide Scale (Cwik *et al*., 2017). Participants rated their agreement with eight statements about suicide (e.g. ‘Everyone has the right to commit suicide’) using a 6-point Likert scale ranging from 0/I disagree to 5/I agree. Items were reverse coded when necessary and a total sum score was calculated with higher scores reflecting more suicide normalisation (Cronbach's *α* = 0.75).

We used the General Help-Seeking Questionnaire (Wilson *et al*., 2005) to measure participants' intentions to seek help when experiencing suicidality from either professionals (items 5 and 7) or family & friends (items 1 to 4). Participants indicated their willingness to seek help from a professional (i.e. psychologist/psychiatrist or general practitioner) or a person from their social network (i.e. partner, friend, parent, other family member) on a Likert Scale ranging from 1/extremely unlikely to 7/extremely likely. We calculated sum scores across items 5 and 7 as well as items 1–4 with higher scores indicating greater willingness to seek help when experiencing suicidality from a professional or from family/friends, respectively.

Current suicidality was measured by one item adapted from the Patient Health Questionnaire-9 (Kroenke *et al*., 2001). Participants rated the extent to which they currently had thoughts that they would be better off dead or of hurting themselves on a Likert scale from 1/not at all to 7/extremely. A current suicidality variable with two categories (1 – not at all; 2–7: at least some) was created.

Suicide literacy was measured by the German version of the 12-item Literacy of Suicide Scale Short Form (LOSS-SF) (Ludwig *et al*., 2022). The scale includes 12 statements with three options to answer (true/false/don't know) covering four domains of suicide-related knowledge, namely causes of suicide, risk factors of suicidal behaviour, signs and symptoms of suicide risk and suicide prevention. Each correct answer was assigned a score of 1, incorrect or ‘don't know’ responses were assigned a score of 0. A total suicide literacy score ranging from 0 to 12 was calculated by summing up all item scores.

Negative mood was measured using the German version of the negative affect subscale of the Positive and Negative Affect Schedule (Breyer and Bluemke, 2016). Participants rated the extent to which 10 adjectives (e.g. ‘upset’) described their current affective state on a Likert scale ranging from 1/not at all to 5/extremely. A total mean score was calculated with higher scores indicating increased negative mood (Cronbach's *α* = 0.87).

We also collected socio-demographic and other relevant personal information including age, gender (male/female/diverse), education level (low: non or vocational education, middle: high school degree, high: university degree), previously experienced suicidality (yes/no; defined as having experienced suicidal thoughts or survived a suicide attempt in the past) and previously received mental health care (yes/no; defined as having received psychiatric or psychotherapeutic treatment in the past).

### Data analysis

Aiming to reduce bias due to insufficient responding effort (Huang *et al*., 2012), we excluded 520 persons who failed to correctly answer one attention check item (‘For this item please select the value to the far right’) or whose response time was below 340 s (cut-off: 10th percentile), leaving 3.269 persons for our analysis. We compared these 3.269 participants to those who failed to meet data quality criteria using t-tests (continuous variables) or chi-square tests (categorical variables). Variable distributions were checked visually and characteristics of the sample were described. We used two linear regression models to investigate associations between suicide stigma and suicide normalisation as well as identify additional determinants, controlling associations for confounding variables (age, gender, education, suicide literacy, previous suicidality, previous mental health care). The associations between suicide stigma and suicide normalisation with help-seeking and current suicidality were tested by several controlled linear and logistic regression models. We used SPSS version 25 for all analyses.

## Results

Based on t-tests and chi-square tests, participants included in the analysis (*N* = 3.269) showed significantly reduced suicide stigma (*M*_diff_ = −0.49, *p* < 0.001) and suicide normalisation (*M*_diff_ = −2.98, *p* < 0.001) as well as higher suicide literacy (*M*_diff_ = 0.31, *p* < 0.01) than those 520 persons who were excluded due to failing data quality criteria. Included participants were also significantly older (*M*_diff_ = 6.28 years, *p* < 0.001), comprised less men (50% *v*. 57%, *p* < 0.01) as well as reported higher percentages of previous suicidality (30% *v*. 22%, *p* < 0.001) and previous mental health care use (31% *v*. 23%, *p* < 0.001) than persons excluded based on data quality criteria.

Participant characteristics are summarised in Table 1. Participants were aged between 18 and 90 years with an average age of 47 years. They were equally split in terms of male and female gender with nine persons identifying as diverse. About 30% of participants indicated previous suicidality (suicidal thoughts and attempts) and about 18% reported current suicidal thoughts. About a third had previously used mental health services. Compared to the midpoint of the scale, participants showed low to medium levels of suicide stigma and suicide normalisation. On average, participants correctly answered about 40% of suicide literacy questions and showed medium levels of intentions to seek help from professionals or family/friends.

**Table 1.** Participant characteristics, *N* = 3.269

Variables (and range of possible scores, where appropriate)	Mean (s.d.) or *n*, %
Suicide stigma^a^ (1–5)	2.00 (0.80)
Suicide normalisation^b^ (0–40)	16.56 (8.02)
Suicide literacy^c^ (0–12)	5.33 (2.32)
Help-seeking from professionals^d^ (2–14)	9.15 (3.52)
Help-seeking from family/friends^e^ (4–28)	16.12 (6.82)
Current suicidality	
No	2670, 81.7%
Yes	599, 18.3%
Age in years (18–90)	46.70 (15.88)
Gender	
Female	1630, 49.9%
Male	1630, 49.9%
Diverse	9, 0.3%
Education	
Low	1100, 33.6%
Middle	1078, 33.0%
High	1091, 33.4%
Previous suicidality (yes)	969, 29.6%
Previous mental health service use (yes)	1.017, 31.1%

a Stigma subscale of the Stigma of Suicide Scale – Short Form.

b Right to commit suicide subscale of the Cognitions Concerning Suicide Scale.

c Literacy of Suicide Scale Short Form.

d Items 5 and 7 from the General Help-Seeking Questionnaire.

e Items 1–4 from the General Help-Seeking Questionnaire.

The links between suicide stigma and suicide normalisation as well as their association with several other potential determinants were investigated using several linear regression models (Table 2). In an uncontrolled model, more suicide stigma was related to less suicide normalisation (*β* = −0.20, *p* < 0.001). Using multiple regression modelling, more suicide stigma was significantly associated with less suicide normalisation (*β* = −0.17, *p* < 0.001), as well as with lower suicide literacy (*β* = −0.22, *p* < 0.001) and younger age (*β* = −0.08, *p* < 0.001). Suicide stigma was higher among men (*β* = 0.20, *p* < 0.001) and lower among persons who reported previous suicidality (*β* = −0.06, *p* < 0.001) or previous mental health service use (*β* = −0.45, *p* = 0.01). More suicide normalisation was significantly associated with less suicide stigma (*β* = −0.17, *p* < 0.001) and older age (*β* = 0.09, *p* < 0.001). Suicide normalisation was higher among men (*β* = 0.08, *p* < 0.001), those with middle or high education as compared to low education (*β* = 0.09, *p* < 0.001 & *β* = 0.12, *p* < 0.001, respectively) and persons who reported previous suicidality (*β* = 0.22, *p* < 0.001).

**Table 2.** Regression models testing the associations between suicide stigma, suicide normalisation and their potential determinants, *N* = 3.269

	Suicide stigma^a^, *R*^2^ = 0.14 (controlled model)				Suicide normalisation^b^, *R*^2^ = 0.10 (controlled model)			
Independent variables	*B*	*β*	*p*	95% CI (*B*)	*B*	*β*	*p*	95% CI (*B*)
Suicide stigma^a^	–	–	–	–	−1.98	−0.20	**<0**.**001**	−2.32 to −1.65
Suicide normalisation^b^	−0.02	−0.20	**<0**.**001**	−0.02 to −0.02	–	–	–	–
Suicide stigma^a^	–	–	–	–	−1.82	−0.18	**<0**.**001**	−2.16 to −1.47
Suicide normalisation^b^	−0.02	−0.17	**<0**.**001**	−0.02 to −0.01	–	–	–	–
Suicide literacy^c^	−0.08	−0.22	**<0**.**001**	−0.09 to −0.06	−0.09	−0.03	0.14	−0.22 to 0.03
Age	−0.01	−0.08	**<0**.**001**	−0.01 to −0.00	0.04	0.09	**<0**.**001**	0.02 to 0.06
Gender								
Female^d^	–	–	–	–	–	–	–	–
Male	0.32	0.20	**<0**.**001**	0.27 to 0.37	1.35	0.08	**<0**.**001**	0.81 to 1.90
Diverse	0.13	0.01	0.60	−0.36 to 0.62	1.44	0.01	0.57	−3.57 to 6.45
Education								
Low^d^	–	–	–	–	–	–	–	–
Middle	−0.02	−0.01	0.66	−0.08 to 0.05	1.51	0.09	**<0**.**001**	0.83 to 2.20
High	−0.07	−0.04	0.06	−0.15 to 0.00	2.10	0.12	**<0**.**001**	1.33 to 2.87
Previous suicidality	−0.10	−0.06	**<0**.**001**	−0.17 to −0.04	3.94	0.22	**<0**.**001**	3.31 to 4.57
Previous mental health service use	−0.08	−0.05	**0**.**01**	−0.14 to −0.02	−0.16	−0.03	0.62	−0.77 to 0.46

a Stigma subscale of the Stigma of Suicide Scale – Short Form.

b Right to commit suicide subscale of the Cognitions Concerning Suicide Scale.

c Literacy of Suicide Scale Short Form.

d Reference category.

p-values that are statistically significant are in bold.

Consequences of suicide stigma and suicide normalisation were investigated using several linear and logistic regression models (Table 3). In uncontrolled models, higher suicide stigma was significantly associated with decreased intentions to seek professional help (*β* = −0.04, *p* = 0.04; *R*^2^ = 0.01), increased willingness to seek help from family and friends (*β* = 0.04, *p* = 0.04; *R*^2^ = 0.01) as well as a reduced probability to experiencing current suicidality (OR = 0.82, *p* < 0.001; Nagelkerke's *R*^2^ = 0.01). After controlling for potential confounding variables, the association between suicide stigma and intentions to seek professional help was no longer significant (*β* = −0.01, *p* = 0.44; *R*^2^ = 0.04), while its' associations with help-seeking from family and friends (*β* = 0.04, *p* = 0.04; *R*^2^ = 0.09), as well as current suicidality (OR = 0.85, *p* = 0.02; Nagelkerke's *R*^2^ = 0.32) remained significant.

**Table 3.** Regression models to determine the effect of suicide stigma and suicide normalisation on intentions to seek professional help for suicidality and current suicidality, *N* = 3.269

	Help-seeking from professionals^a^				Help-seeking from family/friends^e^				Current suicidality		
Independent variables	*B*	*β*	*p*	95% CI (*B*)	*B*	*β*	*p*	95% CI (*B*)	OR	*p*	95% CI (OR)
Suicide stigma^b^	−0.16	−0.04	**0**.**04**	−0.31 to −0.01	0.30	0.04	**0**.**04**	0.00 to 0.59	0.82	**<0**.**001**	0.73 to 0.92
Suicide stigma^b^ (controlled model)^c^	−0.06	−0.01	0.44	−0.22 to 0.10	0.33	0.04	**0**.**03**	0.04 to 0.63	0.85	**0**.**02**	0.74 to 0.98
Suicide normalisation^d^	−0.07	−0.15	**<0**.**001**	−0.08 to −0.05	−0.15	−0.17	**<0**.**001**	−0.18 to −0.12	1.08	**<0**.**001**	1.06 to 1.09
Suicide normalisation^d^ (controlled model)^c^	−0.06	−0.14	**<0**.**001**	−0.08 to −0.05	−0.12	−0.14	**<0**.**001**	−0.14 to −0.09	1.06	**<0**.**001**	1.05 to 1.08

a Items 5 and 7 from the General Help-Seeking Questionnaire.

b Stigma subscale of the Stigma of Suicide Scale – Short Form.

c Model controlled for age, gender, education, suicide literacy, negative mood, previous suicidality and previous mental health service use.

d Right to commit suicide subscale of the Cognitions Concerning Suicide Scale.

^e^Items 1–4 from the General Help-Seeking Questionnaire.

p-values that are statistically significant are in bold.

In uncontrolled models, suicide normalisation was associated with less intentions to seek help from professionals (*β* = −0.15, *p* < 0.001; *R*^2^ = 0.02) or family/friends (*β* = −0.17, *p* < 0.001; *R*^2^ = 0.03) and a greater probability to experiencing current suicidality (OR = 1.08, *p* < 0.001; Nagelkerke's *R*^2^ = 0.08). After controlling for potential confounding variables, suicide normalisation remained significantly associated with less intentions to seeking help from professionals (*β* = −0.14, *p* < 0.001; *R*^2^ = 0.06) or family/friends (*β* = −0.14, *p* < 0.001; *R*^2^ = 0.10), as well as a greater probability to experiencing current suicidality (OR = 1.06, *p* < 0.001; Nagelkerke's *R*^2^ = 0.35).

## Discussion

This study set out to investigate the association between suicide stigma and suicide normalisation as well as to explore their potential determinants and consequences. According to our findings suicide stigma and suicide normalisation are inversely associated, suggesting that interventions to reduce public suicide stigma could unintentionally increase suicide normalisation. This could potentially limit the positive effects of such interventions for suicide prevention due to the detrimental effects of suicide normalisation outlined below. While our cross-sectional findings demand validation by longitudinal and/or randomised studies, they do highlight that more research is needed before interventions targeting public suicide stigma can be developed.

Several factors determined participants' levels of suicide stigma and suicide normalisation. Older age was associated with less suicide stigma but more suicide normalisation, what could be explained by a greater awareness about one's own mortality among older people and increased levels of chronic/incurable illness within their social networks. According to Phillips and Luth (2020), attitudes towards suicide may greatly depend on its circumstances, with more tolerant attitudes when suicide occurs in presence of a chronic or terminal illness. Interestingly, male participants reported elevated levels of both suicide stigma and suicide normalisation, a situation which Eskin *et al*. (2016) described as a ‘fatal trap’ indicating increased suicide risk. Accordingly, high suicide normalisation could push persons in a crisis towards considering suicide as an option while at the same time high suicide stigma may prevent them from seeking support. Indeed, in most countries including Germany, suicide is more common among men than women (WHO, 2019) and our findings suggest that observed high levels of suicide stigma and suicide normalisation among this group may contribute to that. A subgroup analysis among our sample revealed that despite the parallel increase of both suicide stigma and suicide normalisation among men, the inverse association between these two variables was still present among this group. Future research should further investigate this phenomenon and identify reasons for high levels of both suicide stigma and suicide normalisation among men. While more suicide literacy was associated with less suicide stigma in our study, we observed no link between suicide literacy and suicide normalisation. The latter finding is partially in line with one other study (Ludwig *et al*., 2022) that reported a negative association between suicide literacy and the normalisation/glorification of suicidal persons. This initial evidence suggests that interventions to increase suicide literacy could potentially reduce public suicide stigma without increasing suicide normalisation, however more research is needed.

Surprisingly, in contrast to previous studies (e.g. Mayer *et al*., 2020; Oexle *et al*., 2020) suicide stigma was associated with a reduced probability to experience current suicidality among our sample. However, while we investigated associations among members of the general population (and did exclude persons who indicated suicide risk in the screening), previous research typically included high risk groups who had already made experiences with suicidality/suicide, such as persons who survived a suicide attempt (Mayer *et al*., 2020) or those who lost a loved one to suicide (Oexle *et al*., 2020) and investigated their perception of suicide stigma among others rather than participants' personal attitudes. Therefore, we believe that observed differences could be due to both potential social desirability bias (i.e. hesitance to disclose personal stigmatising attitudes) as well as less personal stigmatising attitudes among those with own experiences of suicidality. Additionally, in line with suicide scripts theory (Canetto, 2021) and previous studies (Stack and Kposowa, 2008; Eskin *et al*., 2016; Phillips and Luth, 2020), suicide normalisation was associated with an increased probability to experience current suicidality. Suicide normalisation may therefore reduce the barrier to consider suicide as an option in difficult life situations and should therefore be considered when developing strategies for suicide prevention.

While suicide stigma has been discussed as a potential barrier to seeking professional help, existing studies revealed mixed results and were mostly based on qualitative data (Han *et al*., 2018). For example, while Calear *et al*. (2014) reported a negative association between suicide stigma and intentions to seek professional help among a community sample, another study among Arab youth (Al-Shannaq and Aldalaykeh, 2021) found no association between these two variables. Furthermore, view existing studies observed no link between suicide stigma and intentions to seek help from family and friends (Calear *et al*., 2014; Chan *et al*., 2014; Al-Shannaq and Aldalaykeh, 2021). Among our sample, suicide stigma was not associated with intentions to seek professional help, however we observed a positive link between suicide stigma and intentions to seek help from family and friends. In line with one other study (Phillips and Luth, 2020), suicide normalisation was associated with reduced intentions to seek help from professionals or family and friends. As these partly contrasting study results obtained by us and others may be due to even small differences in study design (e.g. participant selection, used questionnaires, included covariates and their assessment), more quantitative (longitudinal) research using representative samples is needed to pin down how suicide stigma and suicide normalisation may affect help-seeking intentions and behaviours.

Our study is subject to several limitations. As participants were recruited from a pre-established online panel, our data is not fully representative of the German general population despite applying quotas to reflect its composition with regard to age, gender, education and region. Furthermore, to ensure high data quality, we excluded persons who failed our quality criteria, who at the same time also reported increased suicide stigma and suicide normalisation, potentially indicating an increased suicide risk among this group (Eskin *et al*., 2016). However, the extent to which their scores were the result of speedy replies or based on honest data entry could not be determined. To ensure the safety of our participants, persons who indicated increased suicide risk in the screening were excluded from participation, what has implications for the interpretation of our findings. People who previously experienced suicidality could potentially endorse lower levels of suicide stigma and higher levels of suicide normalisation, what would suggest that the observed inverse association between suicide stigma and suicide normalisation could be even higher. Additionally, the reported associations between suicide stigma, suicide normalisation and current suicidality might only be true for those who have not experienced suicidality before. Finally, observed R^2^-values were low and as cross-sectional data was analysed, the directionality of observed effects could not be established.

Past literature suggests that reducing public suicide stigma and thereby improving attitudes towards persons affected by suicide/suicidality is an important aspect of suicide prevention (Hanschmidt *et al*., 2016; Carpiniello and Pinna, 2017; Oexle *et al*., 2020). However, if replicated, our findings would suggest that such interventions could also be harmful when unintentionally normalising suicide. As mentioned in the introduction, the distinction between the two concepts as focusing on attitudes towards people (suicide stigma) *v*. attitudes towards behaviour (suicide normalisation) might be crucial and offer a solution for intervention development. Similarly to initiatives targeting the stigma of alcohol dependence (Schomerus *et al*., 2011), interventions to reduce public suicide stigma should therefore focus on breaking down stereotypes about affected persons and portraying them as individuals in need of support. At the same time, interventions should avoid to convey the message of suicide as a solution to difficult life situations and instead promote forward-looking coping strategies. Indeed, an increasing number of studies suggest that media articles featuring personal stories of coping with and recovering from suicidality can reduce suicide risk and increase help-seeking intentions, a phenomenon called the Papageno-effect (Till *et al*., 2019; Niederkrotenthaler and Till, 2020). Research is needed to investigate the impact of such approaches on suicide stigma and suicide normalisation.

Future research should replicate our findings on the associations between public suicide stigma and suicide normalisation. Furthermore, studies should use longitudinal or randomised data to identify determinants and consequences of these two concepts in order to identify safe targets for interventions to reduce public suicide stigma. Finally, to ensure intervention safety, suicide normalisation should be included as a secondary outcome when evaluating interventions targeting public suicide stigma or suicide literacy.

## Data Availability

Data supporting study findings are available from the corresponding author upon reasonable request.
